# Evaluation of left ventricular dimension and systolic function by standard transthoracic echocardiography before and 24-hours after percutaneous closure of patent ductus arteriosus in 120 dogs

**DOI:** 10.1371/journal.pone.0223676

**Published:** 2019-10-09

**Authors:** Diego Piantedosi, Alfonso Piscitelli, Angela De Rosa, Blanca Serrano Lopez, Marta Claretti, Elisabetta Boz, Laura Mazzoni, Iolanda Navalon Calvo, Paolo Ciaramella, Claudio Bussadori

**Affiliations:** 1 Department of Veterinary Medicine and Animal Productions, University of Naples Federico II, Naples, Italy; 2 Department of Agricultural Sciences, University of Naples Federico II, Naples, Italy; 3 Clinica Veterinaria Gran Sasso, Milano, Italy; 4 Ars Veterinaria, Barcelona, Spain; University of Bari, ITALY

## Abstract

One hundred and twenty dogs were enrolled to value the effect of loading condition changes on left ventricular volumes before and 24-hours after the patent ductus arteriosus (PDA) occlusion by Amplatzer Canine Duct Occluder (ACDO) using standard echocardiography. The animals were divided in pure breed (n. 94) and mixed breed (n. 26); subsequently, the pure breed dogs were divided on the basis of the size of the breed of belonging in 3 groups (small size n. 36; medium size n. 8; large size n. 50). Moreover, the animals were divided in three classes based on their age: until 6 months; 6–12 months; over 12 months. A significant reduction of all the examined parameters (left ventricle internal diameter at end-diastole—LVIDd; left ventricle internal diameter at end-systole—LVIDs; end-diastolic volume—EDV; end-systolic volume—ESV; end-diastolic volume index—EDVI; end-systolic volume index—ESVI; fractional shortening—FS) was observed after ductal closure. Twenty-four hours after the closure, the evaluation of the relative percentage difference (RPD) of the echocardiographic parameters showed a significant reduction, higher in small size breed than in large size breed dogs. No significant difference related to breed size was observed only for RPD_FS variable. A significant interaction effect, between breed size and age classes, was observed only for RPD_EDVI (F = 3.39; p = 0.039). Until six months of age there was no significant difference in RPD_EDVI reduction, but over 6 months a significant reduction between small size and large size breed dogs at 24-hours from the occlusion was observed. In conclusion, our data seem to indicate that small breed dogs show a greater tolerance to congenital volume overload than large breed dogs, and this finding could be justify a delay of PDA closure in order to simplify the interventional procedure.

## Introduction

Patent ductus arteriosus (PDA) is a common congenital cardiovascular disorder in dogs. According to epidemiological studies in dogs, PDA accounts for 21–32% of total congenital heart diseases. Females are more often affected than males with a different breed predisposition [1–2].

PDA represents persistence of the arterial canal that carries blood from the pulmonary artery to the aorta during fetal life, and normally closes within hours after birth in response to hemodynamic and neurohormonal processes [3]. The failure in PDA closure appears to be related to a duct shorter than normal, a hypoplastic duct or a ductal asymmetric muscle mass [4–6].

The persistent and abnormal left-to-right (L-R) shunting through the PDA causes volume overload of the left side of the heart. This leads over time to remodelling in the form of eccentric hypertrophy and dilatation of left ventricle (LV), that represent an appropriate adaptation to the severely increased cardiac output. In untreated patients the persistence of this shunt can lead to pulmonary oedema and clinical signs of congestive heart failure (CHF) [7]. Currently the PDA transcatheter occlusion using the Amplatzer Canine Duct Occluder (ACDO) is considered the gold standard procedure to fix the defect. [8–9].

In dogs, at presentation a reduced LV systolic function is a common finding associated to the volume overload state caused by the PDA [10]. As in humans [11–12], the closure of L-R shunting PDA is followed by preload decrease and afterload increase, resulting in the reduction of the LV size also in dogs [13].

More in detail, the hemodynamic changes before and after PDA closure have been previously described as long-term analysis. The data analysis of long-term outcome in a large canine population indicates that PDA closure determines over time (> 1 year of follow-up) a significant reduction of LV systolic and diastolic diameters when compared with pre-occlusion measures, associated to important survival benefits [14]. Additionally, Stauthammer and colleagues [15] reported the regression of eccentric hypertrophy, and a complete reverse remodelling at 3 months after ductal occlusion in 24 dogs.

Currently, published data regarding short-term changes in cardiac size and function after PDA closure in dogs is limited to studies involving few animals. In a study on 17 dogs PDA closure after 3 days resulted in the decrease of the LV diastolic internal diameter and left atrium/aorta ratio, suggesting an abrupt preload decrease [13]; furthermore, the authors reported a reduction of fractional shortening (FS), explaining this finding as an effect of the sudden changes in loading conditions and not as an impairment of myocardial contractility, according with previous humans studies [11].

At pre-operative evaluation of 34 dogs with PDA Spalla and colleagues [16] have demonstrated that speckle-tracking echocardiography (STE) is able to identify subtle changes in cardiac systolic function and contractility than conventional echocardiography. In a following study, at short-term postoperative evaluation of 25 dogs with PDA the same authors [17] showed no evidence of systolic dysfunction using STE despite the decreases of conventional echocardiographic parameters.

In veterinary literature there is a lack of studies regarding changes in cardiac size and function after PDA closure in relation to the breed size as short-term analysis, and few data is available as long-term analysis. At follow-up over 1 year after PDA closure Saunders and colleagues [14] described increased LV dimension and decreased FS in large breed dogs than small breed dogs.

For the reasons explained above, the aim of this study was to investigate the effect of loading condition changes on LV volumes before and 24-hours after the PDA closure using standard echocardiography in a large population of dogs, considering the size, the age and the breed as possible variables.

## Materials and methods

### Animals

Medical records of client-owned dogs with PDA, referred at private veterinary clinic “Gran Sasso” (Milan, Italy) from 2006 to 2017, were retrospectively evaluated. Using transthoracic echocardiography (TTE), in all dogs PDA was initially visualized by two-dimensional ultrasound, and subsequently the diagnosis was confirmed by color flow mapping. Inclusion criteria were represented by the absence of: 1) other associated congenital or acquired cardiovascular diseases; 2) signs of congestive heart failure (CHF); 3) atrial fibrillation or other arrhythmias; 4) precapillary pulmonary hypertension; 5) reversed (right-to-left) PDA; 6) residual flow after occlusion; 7) complications during ductal occlusion procedure. Animals with mitral valve regurgitation were included if the regurgitant jet was mild, and there was no evidence of valve dysplasia or other morphological abnormalities in the mitral valve apparatus. Animals that received cardiovascular drugs after the PDA diagnosis were excluded from the study.

One hundred twenty dogs completely met the selection criteria and underwent interventional L-R shunting PDA occlusion during the study period. All selected animals were subjected to echocardiography one hour before and 24-hours after the interventional ductal occlusion by ACDO device [18].

As all procedures were performed for diagnostic purposes, the study did not require a consent or ethical approval according to European Directive 2010/63/EU.

### Standard echocardiography

A complete TTE was performed in all dogs placed in right and left recumbency, according with previously published guidelines [19]. Echocardiographic examinations were performed with an Esaote Mylab30Vet, and an Esaote Mylab60, ultrasound machines (Esaote S.p.A., Firenze, Italy) with electronic phased-array transducers ranging from 2 to 10 MHz. The ductus arteriosus was visualized from both the right parasternal short-axis and left cranial parasternal views. Doppler echocardiography was used to confirm L-R shunting through the duct on the initial evaluation and the absence of residual flow after occlusion. During TTE, the cardiac rhythm was monitored using the ECG trace obtained on the screen of the ultrasound machine.

M-mode measurements were obtained from two-dimensional images of the transverse plane of LV at the level of the largest transversal diameter, between papillary muscles and mitral valve, including LV internal diameter at end-diastole (LVIDd) and end-systole (LVIDs). The fractional shortening (FS), as an index of LV systolic function, was calculated by the following formula: [(LVIDd—LVIDs)/ LVIDd] X 100. LV end-diastolic volume (EDV) and end-systolic volume (ESV) were calculated by the Teicholz formula: EDV = (LVIDd^3^ X 7)/(LVIDd + 2.4) and ESV = (LVIDs^3^ X 7)/(LVIDs + 2.4). Values were indexed to the body surface area to obtain the end-diastolic volume index (EDVI) (normal value: < 100 mL/m^2^) and the end-systolic volume index (ESVI) (normal value: < 30 mL/m^2^) [20].

The echocardiograms were performed by cardiology residents and experienced cardiologists and, all the exams were reviewed by an ECVIM board-certified cardiologist (C.B.)

### Statistical analysis

After verifying that the data of echocardiographic variables (LVIDd, LVIDs, EDV, ESV, EDVI, ESVI and FS) were not normally distributed through Shapiro-Wilk test, a 2-tailed Wilcoxon matched-pairs signed rank test was used to examine the differences between pre- and post-occlusion values (baseline and at 24-hours). The significance level for all variables between groups and times was set a priori at *p* ≤ 0.05.

In order to have a proportion of the variation of the echocardiographic parameters (LVIDd, LVIDs, EDV, ESV, EDVI, ESVI and FS) in the pre- and post-occlusion time, we calculated for each variable the relative percentage difference of the post-value with respect to the pre-value.

Relative percentage difference (RPD) variables obtained (RPD_LVIDd, RPD_LVIDs, RPD_EDV, RPD_ESV, RPD_EDVI, RPD_ESVI and RPD_FS) represent the relative change of the echocardiographic parameters, in terms of percentage variation compared to the pre-occlusion values, as result of the percutaneous closure of PDA. In order to verify if the RPD variables followed a normal distribution, we applied Shapiro-Wilk test. After verifying that the percentage difference of each variable was normally distributed, an independent-samples *t* test was performed to assess the significance of the difference between the means of the two canine groups considered, small and large size breed dogs; furthermore, a two-way ANOVA was used to demonstrate the significance of main effects (breed size and age classes of dogs) and their interaction on the RPD variables. A Tukey's honestly significant difference method (Tukey HSD) was used as a post hoc test. Data analysis was performed using R package software [21].

## Results

The selected dogs consisted of 89 females and 31 males. Mean age and weight at the pre-occlusion evaluation were respectively 10.4±6.8 months and 13.8±9.3 kg. Table 1 shows the distribution of canine breeds.

**Table 1:** Distribution of study population for breed and sex.

Breed	Sex		Total
	Male	Female	
German Shepherd	5	12	17
Dobermann	3	6	9
Newfoundland	1	6	7
Jack Russell Terrier	1	4	5
Miniature Poodle	2	3	5
English Cocker Spaniel	0	4	4
Pembroke Welsh Corgi	1	3	4
Maltese	3	1	4
Border Collie	1	2	3
Labrador Retriever	0	3	3
Chihuahua	2	1	3
Smooth-haired Dachshund	1	1	2
Cavalier King Charles Spaniel	0	2	2
White Swiss Shepherd	0	2	2
English Setter	0	2	2
Irish Red Setter	0	2	2
Italian Volpino	2	0	2
West Highland White Terrier	1	1	2
Akita	0	1	1
Australian Shepherd	0	1	1
Old English Sheepdog	1	0	1
Bolognese	1	0	1
Bernese Mountain Dog	0	1	1
Hungarian Short-haired Pointer	0	1	1
Espaneul Breton	0	1	1
Golden Retriever	1	0	1
Blue Gascony Griffon	0	1	1
Czechoslovakian wolfdog	1	0	1
Malinois Belgian Shepherd	0	1	1
Pekingese	0	1	1
Italian Sighthound	1	0	1
Staffordshire Bull Terrier	0	1	1
Italian Short-haired Segugio	0	1	1
Weimaraner	1	0	1
Mixed breed	2	24	26
TOTAL	31	89	120

The animals were divided in pure breed (n. 94) and mixed breed (n. 26); subsequently, the pure breed dogs were divided on the basis of the size of the breed of belonging in 3 groups (small size n. 36; medium size n. 8; large size n. 50), based on breed standards [22].

Considering the group partition of the enrolled animals (small, medium, large size pure breed and mixed breed dogs), the mean age was respectively 10.4±5.7, 9.1±6.4, 9.0±6.7 and 13.7±7.9 months. The mean weight was respectively 5.7±2.7, 14.5±4.9, 20.1±9.3 and 12.4±7.2 kg.

At initial presentation, 91.6% (110/120) of the animals showed an indexed LV end-systolic volume higher than the normal threshold value (ESVI > 30 mL/m^2^), while the indexed LV end-diastolic volume appeared to have increased in 94.1% (113/120) (EDVI > 100 mL/m^2^). Overall, in the study population the statistical comparison with baseline values showed that at 24-hours after ductal occlusion there was a significant reduction in all the examined echocardiographic parameters (LVIDd, LVIDs, EDV, ESV, EDVI, ESVI, FS) (Table 2).

**Table 2:** Echocardiographic variables: Ranks and Wilcoxon signed rank test values.

Variables	Types		N	Mean Rank	Sum of Ranks	Wilcoxon Signed Ranks Test (based on positive ranks)	*p-value*
**LVIDd**	Negative Ranks	(Post<Pre)	114	61.97	7065.00	-9.268	0.000
	Positive Ranks	(Post>Pre)	5	15.00	75.00		
	Ties		1				
**LVIDs**	Negative Ranks	(Post<Pre)	82	62.99	5165.00	-4.444	0.000
	Positive Ranks	(Post>Pre)	36	51.56	1856.00		
	Ties		2				
**EDV**	Negative Ranks	(Post<Pre)	113	62.17	7025.00	-9.162	0.000
	Positive Ranks	(Post>Pre)	6	19.17	115.00		
	Ties		1				
**ESV**	Negative Ranks	(Post<Pre)	83	61.07	5069.00	-4.185	0.000
	Positive Ranks	(Post>Pre)	35	55.77	1952.00		
	Ties		2				
**EDVI**	Negative Ranks	(Post<Pre)	115	62.50	7187.00	-9.315	0.000
	Positive Ranks	(Post>Pre)	5	14.60	73.00		
	Ties		0				
**ESVI**	Negative Ranks	(Post<Pre)	84	62.23	5227.00	-4.610	0.000
	Positive Ranks	(Post>Pre)	34	52.76	1794.00		
	Ties		2				
**FS**	Negative Ranks	(Post<Pre)	100	67.21	6721.00	-8.095	0.000
	Positive Ranks	(Post>Pre)	20	26.95	539.00		
	Ties		0				

Specifically, twenty-four hours after occlusion ESVI decreased within the normal limit only in 10% (12/120) of dogs, while EDVI decreased below the cut-off value in 26.6% (32/120). Furthermore, EDVI and ESVI baseline median values were higher in large size breed dogs than small size breed dogs (EDVI: 210.3 *vs* 178.7 mL/m^2^; ESVI: 81.0 *vs* 55.2 mL/m^2^).

The median pre-occlusion FS value was 33.6% and only in 10.8% (13/120) of animals the value was below of the normal cut-off value. At 24-hours after occlusion 43.3% of dogs (52/120) showed a FS < 25% (normal value: > 25%).

The group of medium size breed dogs was not included in the following statistical analysis due to the small numerical presence, as well as the group of mixed-breed dogs was not included because it was not possible to classify them accurately according to size.

Interestingly, the evaluation of the RPD of the echocardiographic parameters between pre- and post-occlusion showed at 24-hours a significant reduction more high in small size breed than in large size breed dogs. No significant difference related to breed size was observed for RPD_FS variable (Table 3).

**Table 3:** RDP variables: Group of statistics and independent samples *t* test values.

	Size	N	Mean	Std. Dev.	T test (Equal variances assumed)	*p-value*
**RPD_LVIDd**	Small	36	-0.1650	0.10681	-2.520	0.014
	Large	50	-0.1134	0.08302		
**RPD_LVIDs**	Small	36	-0.0733	0.10525	-3.004	0.004
	Large	50	-0.0068	0.09847		
**RPD_EDV**	Small	36	-0.3372	0.19560	-2.742	0.007
	Large	50	-0.2292	0.16861		
**RPD_ESV**	Small	36	-0.1661	0.23353	-3,193	0.002
	Large	50	0.0011	0.24379		
**RPD_EDVI**	Small	36	-0.3404	0.19437	-2,780	0.007
	Large	50	-0.2326	0.16414		
**RPD_ESVI**	Small	36	-0.1704	0.23361	-3,260	0.002
	Large	50	0.0003	0.24386		
**RPD_FS**	Small	36	-0.2208	0.21292	0.448	0.656
	Large	50	-0.2432	0.23982		

Regarding the age the small and large breed dogs were divided in 3 classes, as follows: 1) until 6 months (small breed dogs n. 10; large size breed n. 30); 2) 6–12 months (small breed dogs n. 13; large size breed n. 7); 3) over 12 months (small size breed n. 13; large size breed n. 13).

**Fig 1:**
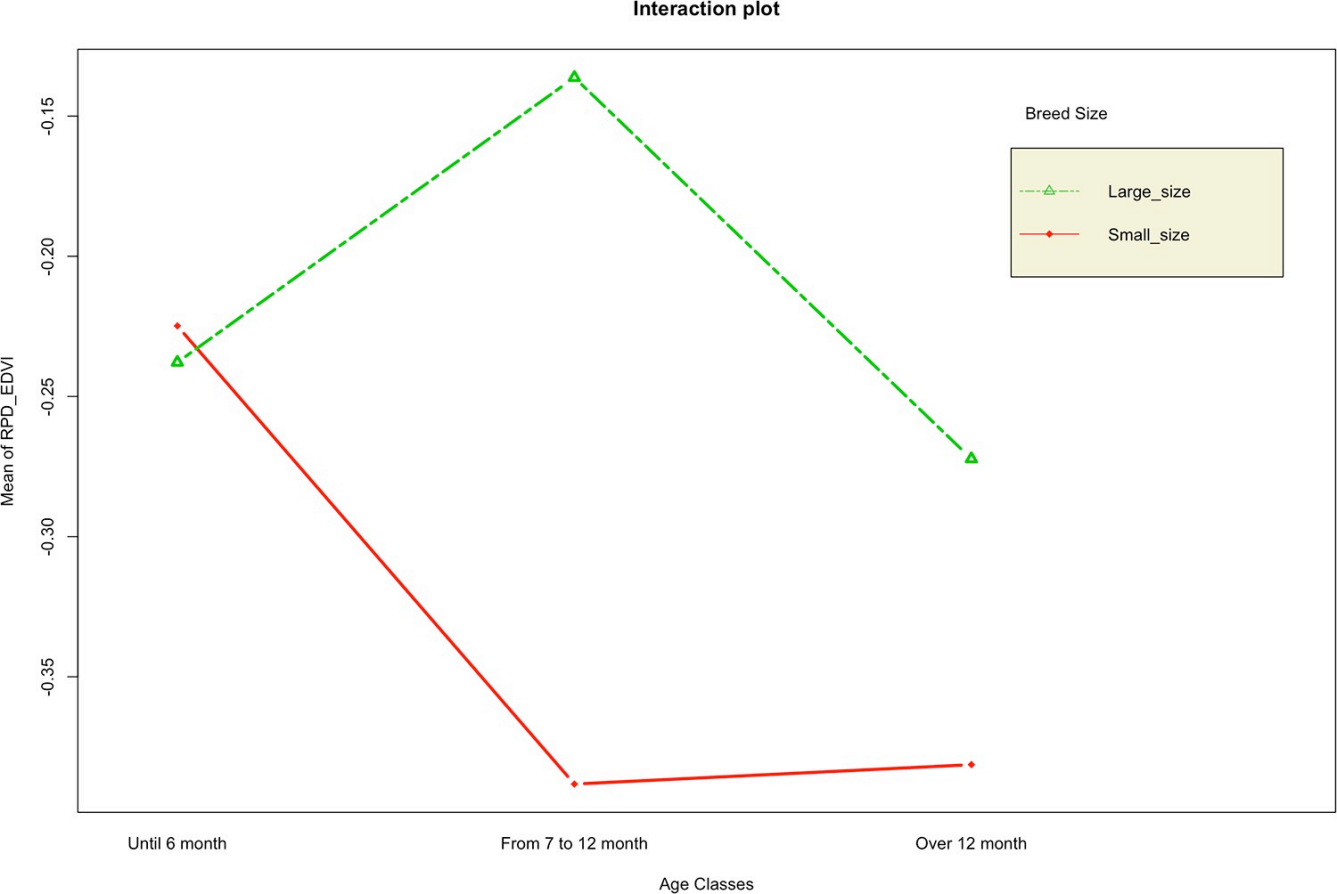
Two-way ANOVA RPD_EDVI profile plot: Interaction between age classes and size.

A statistically significant interaction effect, between breed size and age classes, was observed only for RPD_EDVI (F = 3.39; p = 0.039); with the main effect “breed size” statistically significant (F = 8.205; p = 0.005) and the main effect “age classes” non-statistically significant (F = 1.188; p = 0.31). At 24-hours from the occlusion, RPD_EDVI variable shows a statistically significant mean differences across the breed size factor within the age classes factor. Until six months of age there was no significant difference in RPD_EDVI reduction between small size and large size breed dogs at 24-hours from the occlusion. On the other hand, over 6 months of age a significant reduction of RPD_EDVI was observed in small size than large size breed dogs at 24-hours from the occlusion (Fig 1).

## Discussion

Our study analysed structural and functional left ventricular changes 24-hours after the percutaneous closure of PDA in dogs by using standard TTE. The results show that the ductal closure is associated with the rapid decrease of conventional echocardiographic parameters, represented by LV chamber diameters and volumes, and FS.

The increase in LV dimensions is a consequence of the volume overload secondary to L-R PDA shunting. In this study, increased left ventricular dimensions have been found in dogs with PDA. Our results are in agreement with previous studies in human and veterinary literature [14, 16, 23, 24]. Indeed, the presence of eccentric hypertrophy has been well described in dogs with L-R PDA shunting and is characterized by left-sided chamber dilatation with normal wall thickness, revealing an increased myocardial mass [7, 10, 25]. Increased myocyte proliferation in the form of an eccentric hypertrophy may decrease wall stress by subdividing the load between an increased number of myocytes, which will determine a change of the dimensions of LV, according to the Laplace’s law. The myocardium continues to develop for a short time after birth, and myocyte numbers increase until 6 months postnatally [26]. Probably, the LV of dogs with PDA tolerates better volumetric overload due to an early adaptive response, unlike what happens in other condition of acquired volume overload (e.g. chronic mitral valve disease).

Closure of a PDA is associated with marked change in cardiovascular hemodynamic. The short-term decrease of the end-diastolic LV dimension is explained with the abrupt preload decrease due to the PDA closure. Afterload plays a relative role in the preclosure hemodynamics but gains importance in the postclosure phase, because preload is decreased and afterload is increased by shunt closure [1, 27–29]. Although a more evident decrease in diastolic rather than systolic dimensions generally has been described at short-term evaluation in dogs [13, 15], reflecting the increasing afterload following the PDA closure, in this study a significant reduction of the LV end-systolic dimension at 24-hours post-occlusion has been observed.

It is controversial whether the volumetric overload resulting from the disease may result in LV systolic dysfunction, or on the contrary the ductal closure is associated with the appearance of systolic dysfunction of the LV due to the sudden increase of the afterload. In this canine population only a small portion of dogs (10.8%) had a low baseline FS value (< 25%), according with previous studies that reported FS values in the normal range in dogs with PDA at the time of presentation [13–16]. Saunders and colleagues [14] have reported a significant correlation between increased LV systolic dimensions and systolic dysfunction at initial presentation with persistent heart remodeling at long-term revaluation. Our finding of FS decreasing after PDA closure, with a large part of study population (43.3%) presenting FS below the normal cut-off value, must be referred to the sudden increase of the afterload. As is well known, at first in dogs with PDA an increase in preload will enhance contractility by the Frank-Starling law, which states that an increased venous return will physiologically increase stroke volume, but a long-term volumetric overload could lead in some subjects to LV systolic dysfunction for the persistent wall tension according to the Laplace law, and probably depending on the ductal dimension [24] and the time from birth to diagnosis [14, 30]. This aspect suggests that dogs may experience a worsening of the systolic LV performance assessed echocardiographically after PDA closure, even if this does not necessarily means that an actual impairment of myocardial contractility occurs.

In full-term infants Takahashi and colleague [31] documented a normal LV systolic performance five days after PDA closure, using an independent echocardiographic parameter, as mean normalized systolic ejection rate, that is obtained dividing the ejection fraction (EF) by the ejection time (ET). On the contrary, Nagata and colleagues [32] reported in premature infants a transient deterioration of the LV efficiency, estimated as the ratio of stroke work and pressure-volume area, within 24 hours after PDA ligation, with the recovery to presurgical levels by 2 to 4 days. Recently, in dogs Spalla and colleagues [17] investigated myocardial contractility after PDA closure using STE, an advanced technique that allows to assess myocardial deformation during the cardiac cycle. The postoperative values for longitudinal, radial, circumferential and transverse strain and strain rate were similar to those reported in healthy control dogs.

In this study, the finding of rapid decrease in LV dimensions at 24 hours after PDA closure could be interpreted as a response to the abrupt attenuation of the preload, rather than a sudden regression of eccentric hypertrophy (reverse remodelling). Left ventricle reverse remodelling refers to the possibility of restoring cardiac morphology and function after treatment for a disease condition that has modified LV anatomy [33]. The return to normality of the echocardiographic parameters of LV dimension and function has been reported in infants [28] and in veterinary patients during long-term postoperative follow-up [14, 23, 34], but it is still unclear when LV reverse remodelling begins. In some long-term follow-up studies in dogs with PDA occlusion the appearance over time of acquired cardiac disease (e.g. chronic mitral valve disease) or the persistence of residual ductal flow can explain why the LV dimensions did not return within the reference ranges [14, 23]. Stauthammer and colleguaes [15] reported the regression of eccentric hypertrophy following complete occlusion of uncomplicated PDA in 24 dogs at one-year revaluation. In addition to the LV chamber dimensions, in the study of these latter authors the wall thickness also decreased from the immediate postocclusion state, returning to the preocclusion values, suggesting a reduction in myocardial mass and an effective reverse remodelling.

The results of this study show a different response at 24-hours from the ductal closure in dogs belonging to small- and large-sized breeds. Overall, in small breed dogs there is a greater reduction in indexed ventricular diameters compared to large breed animals. It is possible hypothesize that under PDA loading conditions large breed dogs cope less well wall stress deriving by the increase of the LV transversal diameter according to the Laplace’s law. This speculation is supported by the finding that in our study population EDVI and ESVI baseline values showed median values higher in large size breed dogs. Our findings are in agreement with a previous retrospective study demonstrating that large breed dogs had significantly increased LV dimension and decreased FS at follow-up, in comparison to mixed and small breed dogs [14]. However, further studies in large breed dogs are necessary to clarify whether the different adaptive response to volumetric overload may have long-term consequences on the morphological and functional LV recovery.

The interactional analysis of the age and breed size on the indexed echocardiographic parameters could suggest that in small breed group there is a better tolerance to the parietal stress, and it is more evident after six months of life, occurring a greater LV remodelling according to the Frank-Starling law and to the complete development of pulmonary arterioles; we can hypothesize that this type of patient moves to the right of the Frank-Starling curve more strongly between six months and one year of age, developing an important LV dilatation due to the degree of volume overload, but included in the limits of the preload reserve. Fort this reason, small breed dogs show a rapid reduction of the LV volumes when the preload is reduced by the PDA closure. On the other hand, on the basis of our results we can speculate that in large breed dogs the Frank-Starling mechanism could be already activated since first months of life. These animals in the second semester of life could be excessively shifted to the right in the Frank-Starling curve, with a marked LV remodeling making it more difficult to reduce the transverse diameter at least in the first 24 hours.

The present study presents some limits. The main limit is that the Teichholz equation was the only method used to assess the LV dimensions, although correlation studies have showed a good agreement among different methods, regardless of the different underlying geometrical assumption (Teichholz method, area-length method and allometric scaling) [16].

Another limit was in use of FS as a parameter of systolic function. It is the most used conventional parameter to evaluate systolic LV function, and represent the percentage change in left ventricular chamber dimensions during cardiac cycle. The FS is highly load dependent because it is influenced by changes in ventricular preload, afterload and contractility, as well heart rate. Under modified loading conditions, as occur in PDA, FS can over or under-estimate actual contractility, leading to a misunderstanding of the pathophysiology of the underlined disease. In addition, the M-mode measurements used to calculate FS are recorded from the transverse plane of the left ventricle and only assess transversal movement of the ventricular walls, not accounting for the LV base-apex contraction. A more global assessment of LV systolic function, using two-dimensional methods, such as Simpson’s disk rule and length-area formula, was not possible given the retrospective nature of our study. However, our main purpose in this study was to investigate the effects of parietal stress, and this, in accordance with the Laplace law, is greater where the transverse diameter is greater, and then, consider the dimensional parameters taken from the mono-dimensional echocardiogram of the left ventricle suitable for the purpose.

## Conclusions

In conclusion, the findings of this study seem to indicate that small dogs show a greater tolerance to congenital volume overload induced by PDA compared to large breed dogs. Theoretically, such preliminary data on the effect of the breed size on the adaptive response to PDA could justify, a delay of PDA closure in some small breed dogs allowing weight gain, with the primary aim to simplify the vascular access and the entire interventional procedure (e.g. extremely tiny puppies from small breeds). On the other hand, our data appears to argue against a similar clinical approach in large breed dogs. However, further studies are needed to conclusively prove such hypothesis. Moreover, the behaviour of these and other echocardiographic parameters pre- and post-operatory but even in the long-term follow-up should be further investigated, in order to identify the predictability of some of these regarding the systolic function reserve and the functional remodelling and therefore the postoperative prognosis.

## Supporting information

S1 File Piantedosi et al. 2019 raw data. (XLSX)
